# A Novel Dynamic Update Framework for Epileptic Seizure Prediction

**DOI:** 10.1155/2014/957427

**Published:** 2014-06-22

**Authors:** Min Han, Sunan Ge, Minghui Wang, Xiaojun Hong, Jie Han

**Affiliations:** ^1^Faculty of Electronic Information and Electrical Engineering, Dalian University of Technology, Dalian 116024, China; ^2^Department of Neurology, First Affiliated Hospital of Dalian Medical University, Dalian 116011, China

## Abstract

Epileptic seizure prediction is a difficult problem in clinical applications, and it has the potential to significantly improve the patients' daily lives whose seizures cannot be controlled by either drugs or surgery. However, most current studies of epileptic seizure prediction focus on high sensitivity and low false-positive rate only and lack the flexibility for a variety of epileptic seizures and patients' physical conditions. Therefore, a novel dynamic update framework for epileptic seizure prediction is proposed in this paper. In this framework, two basic sample pools are constructed and updated dynamically. Furthermore, the prediction model can be updated to be the most appropriate one for the prediction of seizures' arrival. Mahalanobis distance is introduced in this part to solve the problem of side information, measuring the distance between two data sets. In addition, a multichannel feature extraction method based on Hilbert-Huang transform and extreme learning machine is utilized to extract the features of a patient's preseizure state against the normal state. At last, a dynamic update epileptic seizure prediction system is built up. Simulations on Freiburg database show that the proposed system has a better performance than the one without update. The research of this paper is significantly helpful for clinical applications, especially for the exploitation of online portable devices.

## 1. Introduction

EPILEPSY is a kind of chronic brain dysfunction syndrome, which is one of the most common serious brain diseases [1]. With a worldwide prevalence of approximately 1%, it affects over 50 million people [2]. Apart from the epilepsy patients whose seizures can be controlled by antiepileptic drugs or the epilepsy surgery, there are still many who cannot be treated sufficiently by any available therapy [3]. These patients are at risk of serious injuries and are prone to acquiring an intense feeling of helplessness that adversely influences their daily lives. Therefore, an effective and reliable seizure prediction method, which can forecast the arrival of seizure, is needed for these patients, providing warning time to allow for safety-enhancing behavioral responses.

The most effective way to predict the arrival of an epileptic seizure is electroencephalogram (EEG) analysis [4]. EEG has been proven to be a kind of nonlinear, nonstationary, and chaotic time series [5], providing information about spatiotemporal patterns of brain electrical activity [6]. Usually, the power spectrum [7], largest Lyapunov exponent [8], correlation dimension [9], similarity index [10], AR coefficients [11], and so forth are calculated to present the features of a piece of EEG recordings, but they are univariate measures. Aarabi et al. pointed out that there was no clear superiority of the nonlinear measures over linear measures, whereas bivariate measures were generally more effective [12]. Therefore, the bivariate measures, such as phase synchronization [13–16], linear correlation [17], and nonlinear interdependence [17], have received close attention from researchers. Since the epileptic seizures are usually characterized by an abnormal synchronized electric discharge of neurons, this paper will extract the EEG features from the point of phase analysis. Considering the limits of Hilbert transform (HT) and wavelet transform (WT) [18], Hilbert-Huang transform (HHT) [19], which is more suitable for nonlinear and nonstationary signal processing, is chosen to calculate the phases of EEG signals. Different from other commonly used phase synchronization indices [20, 21], the phase interaction is quantified by extreme learning machine (ELM) [22].

However, most current studies of automatic epileptic seizure prediction focus on offline methods [7–16, 20, 21]. Although they may have high sensitivity and low false-positive rate temporarily, they cannot keep catching up the patients' changing conditions. Therefore, an adaptive and online method or framework is badly needed. Under the condition that a seizure prediction problem can be solved as a classification problem [23], there are many online classification methods of neural networks [24, 25] though they are not appropriate for this application. In the general online methods, the current samples have a significant effect on the result, and the early samples just have less influence [24, 25]. However, the early samples also play an important role in the application of epileptic seizure prediction, so its importance could not be ignored or reduced. Furthermore, the general online method cannot guarantee the balance of training samples in different classes and easily makes the training bias in favor of one of the classes. Therefore, a novel dynamic update framework is proposed in this paper, which keeps the prediction model fresh through updating the sample pools.

In the proposed framework, distance metric is the key issue, which measures the distance between different sample points or different classes. For instance, *k*-means [26] and *k*-nearest neighbor (KNN) [27] classifiers need to be supplied with a suitable distance metric, through which neighboring data points can be identified. Although Euclidean distance metric is commonly used, it assumes that each feature of data point is equally important and independent of others. This assumption may not be always satisfied in real applications, especially when dealing with high dimensional data where some features may not be tightly related to the topic of interest [28]. Thus, supplying a distance metric is highly problem-specific and determines the success or failure of the learning algorithm or the developed system. In addition, another family of distance metric learning algorithms is developed to make use of pairwise constraints [29–31]. Pairwise constraint is a kind of side information [29]. One popular form of side information is must-links and cannot-links [31]. A must-link indicates the pair of data points must be in the same class, whereas a cannot-link indicates that the two data points must be in two different classes. Another popular form is the relative comparison with “A is closer to B than A is to C” [30]. In this paper, such side information is considered, and Mahalanobis distance is introduced.

All the above considerations motivate our method. Firstly, a novel dynamic update framework for seizure prediction is proposed. Secondly, a basic prediction model based on both multichannel feature extraction and classification is built up and is embedded into the proposed dynamic update framework. Furthermore, an epileptic seizure prediction system is accomplished. The rest of the paper is organized as follows.  Section 2 explains the proposed dynamic update framework for the seizure prediction in detail.  Section 3 interprets the multichannel EEG feature extraction method based on HHT and ELM.  Section 4 outlines the basic prediction model of the epileptic seizure prediction. In Section 5, the performance of the proposed method is evaluated on the Freiburg dataset. Finally, Section 6 ends the paper with some conclusions.

## 2. Dynamic Update Framework for Seizure Prediction Model

**Figure 1:**
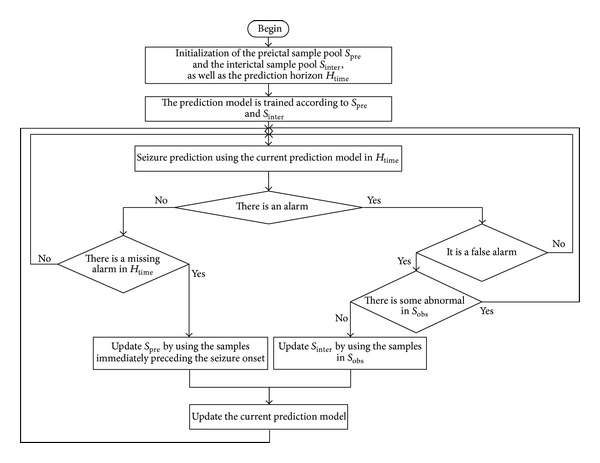
Flow chart of the dynamic update framework for the seizure prediction model.

Currently, most automatic seizure prediction methods are focused on offline methods, of which models cannot be changed and improved once they are built up [7–16, 20, 21]. However, for the reason that the physical conditions of patients always change, if the prediction model can be constructed via only a few seizures and finite interictal recordings, it not only cannot be guaranteed to be the most appropriate one, but also cannot change adaptively to the patients' health conditions. Therefore, training the seizure prediction model dynamically is necessary. Based on the abovementioned, a novel dynamic update framework for the seizure prediction is proposed, which can achieve the purpose of self-adaptive. It is used to update the training dataset.  Figure 1 shows the flow chart of the dynamic update framework.

For each of the patients, there are datasets called ictal and interictal. The ictal periods, which contain epileptic seizure period and preictal period, are determined based on identification of typical seizure patterns preceding clinically manifest seizures in intracranial recordings by visual inspection of experienced epileptologists. Herein, for acquiring enough training samples, the preictal period is at least 50 min. It can be seen from Figure 1 that two sample pools, *S*_pre_ and *S*_inter_, need to be built up for the dynamic update framework first, which are filled with preictal samples and interictal samples, respectively. The prediction model is built up based on *S*_pre_ and *S*_inter_, and the prediction horizon *H*_time_ is initialized. The system uses the current model to predict the seizures in the *H*_time_. Once a false alarm occurs, whether the sample set *S*_obs_ (it will be explained in Section 2.2 ) is abnormal or not needs to be decided, and only the normal samples can be used to update the interictal sample pool *S*_inter_ of the model. If the seizure cannot be predicted (i.e., the seizure alarm missed), the preictal sample pool *S*_pre_ is updated by using the samples of 30 to 40 minutes immediately preceding the seizure onset. At last, the prediction model can be updated based on the new sample pools, and the system can use the new prediction model to predict seizures.

In the above procedures, three parts need to be discussed and explained. They are the abnormal detection, the criterion of the sample pools' update, and the two conditions for update. For the abnormal detection, a criterion needs to be decided to determine what kind of sample is abnormal. For the criterion of the sample pools' update, a rule needs to be decided to determine how the old samples are replaced by the new samples. At last, two conditions, false alarm and missing alarm, are considered.

Currently, the commonly used distance metrics are Euclidean distance [32], Mahalanobis distance [28], Manhattan distance [33], Chebyshev distance [34], and so on. Obviously, Manhattan distance and Chebyshev distance are not appropriate for the problem in this paper according to their theories. The Euclidean distance metric assumes that each feature of data point is equally important and independent of others. This assumption may not be always satisfied in real applications, especially when dealing with high dimensional data where some features may not be tightly related to the topic of interest [28]. However, the Mahalanobis distance is measured between two data points or two data sets in the space defined by relevant features [28]. Since it accounts for unequal variances as well as correlations between features, it will adequately evaluate the distance by assigning different weights or importance factors to the features of data points. Only when the features are uncorrelated, the distance under a Mahalanobis distance metric is different from that under the Euclidean distance metric. In addition, geometrically, a Mahalanobis distance metric can adjust the geometrical distribution of data so that the distance between similar data points is small. Therefore, Mahalanobis distance is an effective metric to measure the similarity of two sample sets, and so it is used in this paper in both the abnormal detection and the update of the sample pools.

In what follows [35], given *x*_1_ and *x*_2_ are two points of the observed dataset *X*, their Mahalanobis distance can be calculated as follows:

(1) $$\begin{array}{c} M_{X}\left(x_{1},x_{2}\right)=\sqrt{{\left(x_{1}-x_{2}\right)}^{T}\Sigma^{-1}\left(x_{1}-x_{2}\right)}. \end{array}$$

The Mahalanobis distance of a point *x* and the set *X* can be calculated as follows:

(2) $$\begin{array}{c} M\left(x,X\right)=\sqrt{{\left(x-\mu \right)}^{T}\Sigma^{-1}\left(x-\mu \right)}=M_{X}\left(x,\mu \right), \end{array}$$

where *μ* and Σ are the mean and covariance matrix of the observed dataset *X*.

### 2.1. Abnormal Detection

Suppose the interictal sample set is *S* and the Mahalanobis distances are calculated between *S* and *S*_pre_ and between *S* and *S*_inter_, which are denoted by *M*(*S*, *S*_pre_) and *M*(*S*, *S*_inter_), respectively. If *M*(*S*, *S*_pre_) is less or equal to *λ* · *M*(*S*, *S*_inter_), the samples in *S* are taken as abnormal, which is shown as follows:

(3) normal  sample,if  M(S,Spre)>λ·M(S,Sinter),abnormal  sample,else,

calculated according to (1) and (2).

### 2.2. Criterion of the Sample Pools' Update

The idea of support vector is introduced in [11], and the farthest sample from the support vector will be replaced. Suppose there are *N*_1_ samples in the sample pool *S*_pre_, *N*_2_ samples in the sample pool *S*_inter_, and *N*_3_ samples in the sample set *S*_obs_ to be observed or filled with the samples immediately preceding the seizure onset which misses alarm.

The update of the interictal sample pool *S*_inter_: calculate the Mahalanobis distances *M*(*S*_inter_ ∪ *S*_obs_, *S*_pre_), and sort the *N*_2_ + *N*_3_ samples in *S*_inter_ ∪ *S*_obs_ according to the above Mahalanobis distances. Only the first *N*_2_ samples in *S*_inter_ ∪ *S*_obs_ will be retained as the new sample pool *S*_inter_.

The update of the preictal sample pool *S*_pre_: calculate the Mahalanobis distances *M*(*S*_pre_ ∪ *S*_obs_, *S*_inter_), and sort the *N*_1_ + *N*_3_ samples in *S*_pre_ ∪ *S*_obs_ according to the above Mahalanobis distances. Only the first *N*_1_ samples in *S*_pre_ ∪ *S*_obs_ will be retained as the new sample pool *S*_pre_.

### 2.3. Two Conditions for Update

Suppose the current window is win, the dealt-with window is win-pre, and the relationship of time is win-pre + *H*_time_ = win. Both the two windows are corresponding to the observation window, which will be explained in Step 4 in Section 4. The main idea is that the window win-pre is dealt with according to the state of window win.

**Figure 2:**
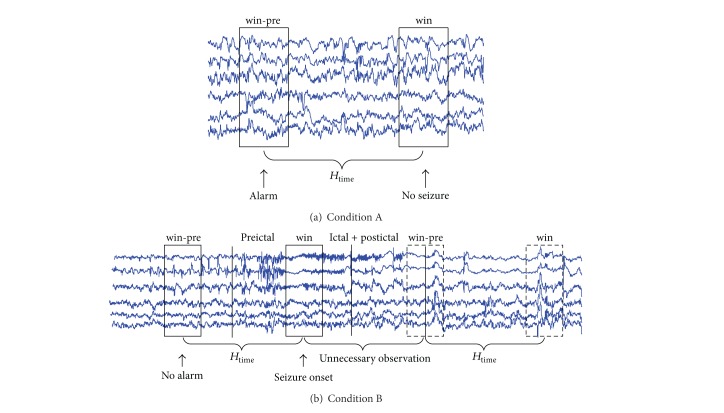
Two conditions for update. Condition A is for false alarm and condition B is for missing alarm.

*Condition A (seen as Figure 2(a)).* It is a false alarm condition when there is not seizure onset actually in the window win while there is an alarm in the window win-pre. Firstly, the samples in the window win-pre are decided whether they are abnormal or not. Then, the interictal sample pool *S*_inter_ will be updated using the samples in the window win-pre if they are not abnormal. 

*Condition B (seen as Figure 2(b)).* It is a missing alarm condition when a seizure dose not alarm in the window win. Firstly, the preictal sample pool *S*_pre_ will be updated using the samples of a period of time immediately preceding the window win. And then, the EEG recordings will be going on observed from the time point of “ictal + postictal + *H*_time_.”

## 3. Multichannel EEG Feature Extraction Based on HHT and ELM

Although the exact mechanisms underlying seizure generation are still uncertain, more and more studies show that epileptic seizures are usually characterized by an abnormal synchronized electric discharge of neurons involved in the epileptic process [36], implying that a method based on phase analysis should be adopted. Phase synchronization method is popular in EEG analysis, using some indices to represent the degree of phase synchronization [13–16]. However, the information provided by these indices is simple, and it is limited to double-channel analysis. Thus, phase synchronization method becomes increasingly important to explore a multivariate one for EEG analysis.

In the phase analysis method, there are two key points to focus on: phase calculation and phase interaction information extraction. Firstly, HT [13] and WT [15] are usually adopted to calculate the phases of signals. But, there are some drawbacks of them. On the one hand, HT computes the instantaneous amplitude, frequency, and phase of the signals using the mathematics framework in macroperspective, and it is likely that negative frequency occurs. On the other hand, a proper wavelet needs to be selected for WT, and also its transformed result is not unique [18]. Secondly, the indices for quantifying the phase interaction are limited to double-channel analysis, which extracts features among multiple bivariate channels and does not represent the useful information that is available among all channels [20, 21].

**Figure 3:**
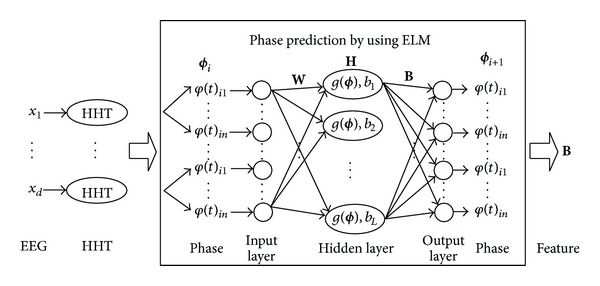
Structure of the multichannel EEG feature extraction based on HHT and ELM.

According to the above considerations, a novel multichannel EEG feature extraction method based on HHT and ELM is utilized in this paper, which is named HHT-ELM for short. In general, HHT and ELM network take place of the phase synchronization indices (such as mean phase coherence (MPC) [13]) at the same time. HHT is a kind of nonlinear and nonstationary signal processing method, which decomposes and transforms adaptively according to the data itself [19]. ELM is utilized for imitating and identifying the phase interaction information among all channels with a low computation cost.  Figure 3 shows the main structure of HHT-ELM.

As is shown in Figure 3, the inputs of the whole structure are EEG recordings with *d* channels which are preprocessed by the filter. They are transformed into phase series by HHT. Then, ELM network is used to process the phase series. Through nonlinear mapping and one-step prediction training, the output weights **B** of ELM are obtained, which are taken as the EEG features we need. In the following sections, the two main parts of HHT-ELM will be explained in detail.

### 3.1. HHT for Phase Calculation

This section presents the HHT method in a nutshell. All the details regarding the implementation of HHT algorithm and Matlab codes are fully available in [37]. Empirical mode decomposition (EMD) algorithm is the basis of HHT, which was proposed by Huang et al. in 1998 [19]. It is a new method applicable for time-frequency analysis of nonstationary and nonlinear time series. The feature of EMD is time series smoothing processing; that is, the different scales of fluctuations or trends of the upcoming complex signals are decomposed gradually. A group of linear and steady-state data sequences with different characteristic time scales is obtained using EMD, and each sequence is taken as an intrinsic mode function (IMF) [38]. IMFs are obtained through the so-called “sifting process,” and they must meet the following two criteria: (1) the number of local maxima and the number of local minima must differ by at most one; (2) the mean of its upper and lower envelopes must equal zero [39].

Given an original signal *x*(*t*), EMD can be summarized as follows, including the “sifting process” [40]:

 Step 1: identify all the extremes of *x*(*t*);
 Step 2: interpolate between minimums (or maximums), ending up with envelope *e*_min⁡_(*t*) (or *e*_max⁡_(*t*));
 Step 3: compute the mean *m*(*t*) = [*e*_min⁡_(*t*) + *e*_max⁡_(*t*)]/2;
 Step 4: extract the detail *d*(*t*) = *x*(*t*) − *m*(*t*), and iterate Steps 1 to 4 until *d*(*t*) meets the criteria of IMF;
 Step 5: denote *d*(*t*) as imf_1_, and compute the residual function *r*(*t*) = *x*(*t*) − imf_1_;
 Step 6: iterate Steps 1 to 5 on the residual function *r*(*t*) until *r*(*t*) is a monotonic function.

Thus, the original signal *x*(*t*) can be decomposed into

(4) $$\begin{array}{c} x\left(t\right)=\overset{n}{\underset{i=1}{\sum }}{\text{imf}}_{i}\left(t\right)+r\left(t\right), \end{array}$$

where *n* denotes the number of IMFs and *r*(*t*) is called the residual function, representing the trend of signal *x*(*t*).

From the above steps, it can be seen that the underlying principle of EMD is to locally identify the most rapid oscillations in the signal, which are defined as waveforms interpolating interwoven local maximum and minimum. To do so, the local maximum points (resp., the local minimum points) are interpolated with a cubic spline, to determine the upper (resp., the lower) envelope. The mean envelope is then subtracted from the original signal, and the same interpolation scheme is reiterated on the remainder. The “sifting process” terminates when the mean envelope is reasonably zero everywhere, and the resultant signal is designated as the first order IMF. The higher order IMFs are iteratively extracted applying the same procedure to the original signal, after removing the previous IMFs [38]. In all cases, IMFs can be viewed as a nonlinear frequency narrowband, from high frequency to low frequency. For different signals, EMD has the ability of adaptive decomposition and the decomposition result is unique.

Based on EMD, HHT can be explained as follows. HHT consists of EMD and HT [19, 41]. For given signal *x*(*t*), according to (4), EMD can decompose *x*(*t*) into a group of IMFs, imf_*i*_  (*i* = 1,2,…, *n*), where *n* is the number of IMFs. Then, applying HT to the IMF components, the following is obtained:

(5) $$\begin{array}{c} Z\left(t\right)=\text{imf}\left(t\right)+iH\left[\text{imf}\left(t\right)\right]=a\left(t\right){\text{e}}^{i\int w(t)dt}, \end{array}$$

where

(6) a(t)=imf2(t)+H2[imf(t)],φ(t)=arctan(H[imf(t)]imf(t)),w(t)=dφ(t)dt.

The instantaneous angle frequency *w*(*t*) and amplitude *a*(*t*) of IMF can be obtained. A time-frequency distribution for signal *x*(*t*) is obtained.

Comparing with other commonly used transform methods, HHT is more suitable for handling the nonlinear, nonstationary signal processing. It decomposes and transforms adaptively according to the data itself and does not require a specific decomposition base.

### 3.2. ELM for Phase Interaction Quantization

After calculating the phase, the phase interaction information needs to be extracted. Currently, MPC is mostly employed to assess the degree of phase synchronization [13–16, 20, 21] (the definition of MPC can be found in these references), but it contains limited information of phase synchronization and may leave out some important information which is propitious to present the complete characteristic. Therefore, a new method is proposed to deal with multichannel and extract out all the useful phase interaction information among all channels. Neural networks are employed to replace the index functions. By means of one-step prediction of the phases, the signal system can be identified.

As is mentioned before, the feature extraction method needs to be fast, so that it can be used in online device. However, the general neural networks usually iterate to calculate the output weights and need to design the input weights and biases at the same time, which takes high computation cost [22]. Consequently, ELM is used, which has been demonstrated to have impressive performance in regression and classification tasks due to its high generalization ability and fast learning speed. Comparing with the traditional neural networks and SVM, ELM not only has a high accuracy in much shorter training time, but also can avoid the problems such as overfitting, local minima, and improper learning rate. Moreover, ELM works with no iteration and least human intervention [22]. The principle of ELM is explained next, which works for single-hidden layer feedforward networks (SLFNs).

Let {**s**_*i*_, **o**_*i*_}_*i*=1_^*N*^ be a set of arbitrary instances, where **s**_*i*_ = [*s*_*i*1_, *s*_*i*2_,…, *s*_*in*_]^*T*^ ∈ **R** ^*n*^ is the *i*th input and **o**_*i*_ = [*o*_*i*1_, *o*_*i*2_,…, *o*_*im*_]^*T*^ ∈ **R** ^*m*^ is the *i*th target output. If there exists a standard SLFN with *L* hidden neurons able to approximate the *N* instances (**s**_*i*_, **o**_*i*_), *i* = 1,2,…, *N* with zero error, then it can be mathematically modeled by the following equation:

(7) $$\begin{array}{c} \overset{L}{\underset{i=1}{\sum }}\beta_{i}g\left(w_{i}\cdot s_{j}+b_{i}\right)=o_{j},\;j=1,\ldots ,N, \end{array}$$

where **w**_*i*_ = [*w*_*i*1_, *w*_*i*2_,…, *w*_*in*_]^*T*^ denotes the weight vector connecting the *i*th hidden neuron and the input neurons, **β**_*i*_ = [*β*_*i*1_, *β*_*i*2_,…, *β*_*im*_]^*T*^ denotes the weight vector connecting the *i*th hidden neuron and output neurons, *b*_*i*_ represents the bias of the *i*th hidden neuron, and *g*(·) is the activation function. Equation (7) can be expressed as follows:

(8) H(w1,…,wL,b1,…,bL,s1,…,sN) =[g(w1·s1+b1)⋯g(wL·s1+bL)⋮⋱⋮g(w1·sN+b1)⋯g(wL·sN+bL)],

(9) $$\begin{array}{c} B={\left[\begin{array}{ccc} \beta_{1}^{T} & \cdots & \beta_{L}^{T} \end{array}\right]}^{T}, \end{array}$$

(10) $$\begin{array}{c} O={\left[\begin{array}{ccc} o_{1}^{T} & \cdots & o_{N}^{T} \end{array}\right]}^{T}. \end{array}$$

**H** is the hidden layer output matrix of SLFN. The input weights **w**_*i*_ and the hidden layer bias *b*_*i*_ are generated randomly. The processing train of a SLFN is to discover a least-squares solution **B** of the linear system **H** **B** = **O**. **B** = **Η** ^†^ **O** is the best weight matrix, where **Η** ^†^ is the Moore-Penrose generalized inverse. ELM utilizes such a Moore-Penrose inverse approach. It can perform at extremely fast learning speed. Unlike some conventional methods, for example, backpropagation (BP) algorithm, ELM is able to avoid the problems in tuning control parameters (learning epochs, learning rate, and so on) and keeping to local minimum.

The procedures of ELM are expressed as follows.

 Step 1: Choose arbitrary value for input weights **w**_*i*_ and biases *b*_*i*_ of hidden neurons.
 Step 2: Calculate hidden layer output matrix **H** according to (8).
 Step 3: Obtain the optimal **B** using **B** = **Η** ^†^ **O**.

By means of ELM, the phase interaction can be quantified. Because the research of this paper is based on a moving-window analysis, the feature extraction method acts on each time window. In real line box of Figure 3, the input layer of ELM is phase **ϕ**_*i*_, and the output layer of ELM is phase **ϕ**_*i*+1_. In each time window, the one- step prediction training procedure of ELM is used to fit the actual phase series. Then, the output weights of ELM are obtained, which are taken as the useful extracted EEG features of the corresponding time window. The features contain the information of the phase interaction among all channels.

## 4. Basic Epileptic Seizure Prediction Model

**Figure 4:**
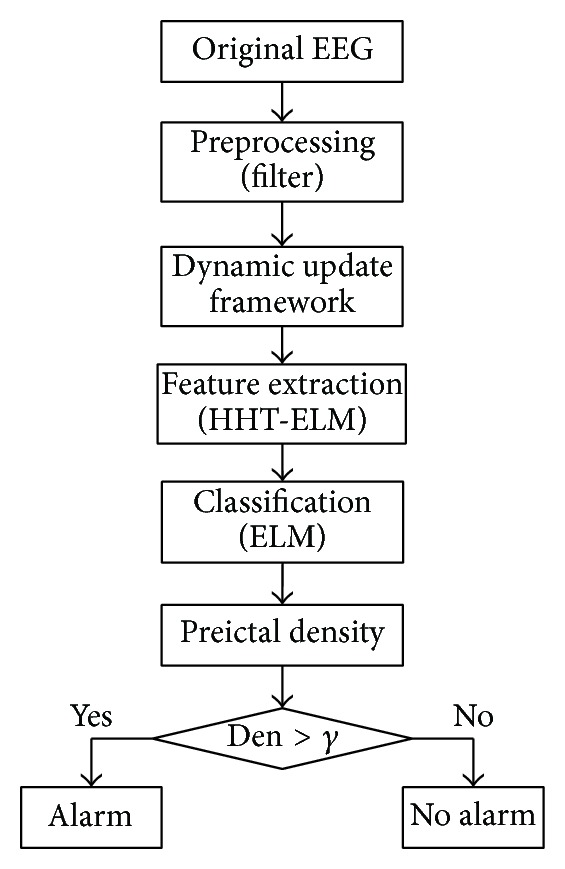
Flow chart of the basic epileptic seizure prediction based on HHT-ELM feature extraction. Den denotes the “preictal density” and *γ* denotes the density threshold.

This section realizes a system that is able to predict the arrival of an epileptic seizure.  Figure 4 reveals the basic flow chart of it, whose interpretations will be explained as follows.

**Step 1 (preprocessing).**

The EEG signal is affected by a superimposed sinusoidal disturbance at the frequency of the ac power supply. In order to eliminate the influence of such a disturbance, a 50 Hz band-suppression filter is exploited in this step. This choice aims at preserving the available information as much as possible in the EEG recordings.

**Step 2 (dynamic update framework).**

This step focuses on constructing the preictal sample pool and the interictal sample pool for next step of feature extracted. The data is continual update to achieve the optimal prediction model. The detailed processing is described in Section 2.

**Step 3 (feature extraction).**

The EEG signals that have been acquired by the dynamic update framework are passed through the feature extraction step, producing a feature vector to be used for classification. Feature extraction is done using the data over time windows. In this paper, the feature extraction method HHT-ELM is adopted.

**Step 4 (classification).**

Following the feature extraction, ELM is used to learn the mappings from the training set features into the patient's state: preictal or interictal. In this way, the seizure prediction problem can be converted into a binary classification one. The output of this step is a binary variable which should be set equal to 1 whenever the segment of EEG is a preictal state and equal to 0 in an interictal state.

The time taken to train the classification models should be an important factor of developing online portable devices for epileptic seizures, because the devices will need to update their training during use. However, the classifiers with high accuracy often cannot meet the demand of speed. In our study, we investigate the usage of ELM to obtain a balance between high classification accuracy and short training time [22].

**Step 5 (calculation of “preictal density”).**

The final stage of the system is to calculate the “preictal density.” From the classification results, the trend of patient's brain condition can be found. However, EEG is a kind of nonstationary signal and can easily be interfered by some factors; therefore, the classification results must have much noise. In fact, when observing the output obtained by ELM, a chattering behavior can often be found. In order to avoid this phenomenon which negatively affects the seizure prediction capability, the following “preictal density” Den in an observation window win_os_ is calculated:

(11) $$\begin{array}{c} \text{Den}=\frac{N_{\text{preictal}}}{N_{\text{preictal}}+N_{\text{interictal}}}, \end{array}$$

and a density threshold *γ* should be chosen. As Figure 4 shows, when Den is over *γ*, an alarm is produced, otherwise no alarm.

## 5. Experimental Results

### 5.1. EEG Database

To evaluate the proposed method, some simulations on the Freiburg EEG database (http://epilepsy.uni-freiburg.de/) are carried out. The database contains invasive EEG recordings of 21 patients suffering from medically intractable focal epilepsy [42].

The EEG data were recorded during invasive presurgical epilepsy monitoring at the Epilepsy Center of the University Hospital of Freiburg, Germany. In order to obtain a high signal-to-noise ratio, fewer artifacts, and to record directly from focal areas, intracranial grid, strip, and depth electrodes were utilized.

The EEG data were obtained using a Neurofile NT digital video EEG system with 128 channels, 256 Hz sampling rate, and a 16-bit analogue-to-digital converter. The 6 contacts of all implanted grid, strip, and depth electrodes were selected by visual inspection of the raw data by a certified epileptologist. Three of them were chosen from the seizure onset zone, involved early in ictal activity. The remaining three electrodes were selected as not involved or involved latest during seizure spread.

For each of the patients, there are datasets called ictal and interictal. The former contains files with epileptic seizures that were at least 50 min preictal data, and the latter contains approximately 24 h of EEG recordings without seizure activity. At least 24 h of continuous interictal recordings is available for 13 patients. For the remaining patients, interictal invasive EEG data consisting of less than 24 h were joined together, to end up with at least 24 h per patient. The ictal periods were determined based on the identification of typical seizure patterns preceding clinically manifest seizures in intracranial recordings by visual inspection of experienced epileptologists.

For evaluating the performance of dynamic update method, it needs enough testing sample for reflecting the reasonable function of dynamic update model. Considering the characterization of machine learning, in our study, only the 9 patients in the database are used, whose seizure numbers are all 5. The seizure occurrence period is different for each individual patient. Most of them have a short seizure occurrence period of a few minutes. A maximum seizure occurrence period is 28.5 min for all patients. The details of the 9 patients' characteristics are listed in the Appendix.

### 5.2. Simulations

All the simulations were based on a 1.80 GHz 2-core CPU with 2.00 GB memory. In order to show the effectiveness of the proposed method, both the experiments of dynamic update and no update for the model were carried out. The comparison of them was shown as follows.

The initial preictal sample pool *S*_pre_ and the interictal sample pool *S*_inter_ were generated for each patient separately. For the preictal sample pool *S*_pre_, the first two seizures were used. By using the intervals of 10 s and overlapped them by 50%, 37.6 minutes of data immediately preceding each seizure can produce 450 preictal samples. For the interictal sample pool *S*_inter_, the interictal training samples are also generated using the intervals of 10 s, randomly chosen from the interictal recordings of 24 h for a total of 150 minutes, that is, 900 interictal samples.

The implementation of the proposed method also requires the choice of some design parameters. The time window is set at 10 s from experience (since the EEG data is 256 Hz sampling rate; therefore, there are 2560 sample points for each time window correspondingly), and in order to avoid the edge effect, the time window is overlapped by 50%. For HHT-ELM, the maximum number of IMFs is set at 3, so that the number of IMFs is limited, which is convenient to the operation of the feature extraction and classification procedures. In addition, the number of hidden neurons of ELM is empirically determined as 10, and the sigmoid function is chosen as the activation function. As to ELM [43] for classification, the number of hidden neurons is set at 1000, and the activation function uses sigmoid functions. The observation window win_os_ is 1.5 min, and the density threshold *γ* is 0.7. For the dynamic update framework, the prediction horizon *H*_time_ is set at 110 min, and the parameter *λ* is set at 1.

### 5.3. Evaluations and Results

In order to illustrate the results clearly, the following evaluations are used: the sensitivity *s*_*e*_, the false-positive rate fpr, the advance prediction time *t*_*a*_, and the performance index *P*. The sensitivity *s*_*e*_ is the percentage of seizures which have been predicted accurately. The false-positive rate fpr is defined as the number of false alarms per hour in interictal EEG. The advance prediction time *t*_*a*_ is defined as the difference between the seizure beginning time marked in the database and the alarm time determined by the prediction system. In reality, the sensitivity cannot be focused on only, and a bad false-positive rate always brings troubles for clinical applications. In clinical analysis, the predict sensitivity and false-positive rate are both the most important evaluation indicators of the seizure prediction. Only when both of them reach the best balance point, the prediction system is satisfactory. Therefore, the prediction system needs to be evaluated via both indicators, and a performance index *P* is employed [14, 16], which combines the two indicators together as defined in the following:

(12) $$\begin{array}{c} P=\sqrt{\frac{\left({\bar{s}}_{e}^{2}+{\bar{s}}_{p}^{2}\right)}{2}}, \end{array}$$

where ${\bar{s}}_{e}$ denotes the mean sensitivity and ${\bar{s}}_{p}$ denotes the specificity rate, which is defined as 1 minus the mean false-positive rate for the entire group of patients (when fpr is more than 1 h^−1^, ${\bar{s}}_{p}$ is set at zero). Therefore, the larger *P* is, the better the performance of the system is.

Based on the above methods, 9 patients who have 5 seizures recordings totally are chosen as the simulation objects. And Tables 1 and 2 give the results.

In Table 1, “0” presents there is no alarm in the column of “Advance time.” It can be seen from Table 1 that the dynamic update for the model method is more effective. From the point of sensitivity, each patient's sensitivities of the two methods are the same except patient 17. For patient 17, the sensitivity is 66.7% of the method with dynamic model update whereas it is 33.3% of the method without model update. For the situation without model update, only the first seizure can be detected, and the false-positive rate is high, 0.38 h^−1^. However, for the situation with dynamic model update, the first and the third seizures can be detected, and the false-positive rate is much lower, 0.14 h^−1^. Therefore, we can conclude that the sample pools become more diversified and the prediction model becomes closer to the current physical conditions. From the point of false-positive rate, patients 4, 5, 9, 10, 16, and 20 are separately almost the same of the two methods, whereas they are significantly different from patients 17, 18, and 21. For patients 17, 18, and 21, the false-positive rates of the method with dynamic model update are much lower than the method without update. The model can change with the patient's physical condition all the time and such update keeps it being closer to the reality as possible.  Table 2 lists out the mean results of Table 1, and the performance index *P* shows that the method with dynamic model update performs better than the method without update.

For the shared Freiburg data, a lot of attempts have been made to predict epileptic seizures, all with a varying degree of success. Some research used nonlinear measures including the dynamic similarity index with MPC [44, 45], the wavelet-based nonlinear similarity index [46], and the lag synchronization index with MPC [47]. By using single bivariate feature of [45], the average seizure prediction sensitivity achieved 35.2% and 43.2% with “OR” and “AND” combination system, respectively, when SOP (seizure occurrence period) is 30 min under a maximum false prediction rate of 0.15 h^−1^. Averaged sensitivity values of 60% were obtained for fpr of 0.15 h^−1^ by replacing dynamic similarity index with lag synchronization index in [47]. Compared with [45, 47], the dynamic update method achieved the larger mean sensitivity (85.2%) and lower mean fpr (0.04 h^−1^) by a multichannel EEG feature extraction method.

In a more recent research, in order to enhance the sensitivity, a set of quantitative univariate and bivariate nonlinear features [48] were used in seizure prediction. For patients 5, 9, 17, 18, 20, and 21, a relatively high sensitivity of 88.83% with an average fpr of 0.13 h^−1^ was got by means of the system of [48] under a SOP of 50 min. Further, the machine learning was introduced for making massive efforts to improve the sensitivity and fpr. In [49, 50], they, respectively, acquired the sensitivity of 88.89% and 95.56% for patients 4, 5, 9, 10, 16, 17, 18, 20, and 21. The average fpr of them were, respectively, 0.096 h^−1^ and 0.22 h^−1^. Comparing with the above said methods, for dynamic update method, although the sensitivity was lower than other methods, however, the mean fpr still gained the best results. The aim of dynamic update framework is to reduce the false prediction rate without needing to set a maximum false alarm condition. By calculating the performance index *P* for reported results of [5–7], the *P* values of them were, respectively, 0.8784, 0.8713, and 0.8965. It clearly found that the dynamic update method outperformed other methods in terms of the performance index *P*.

## 6. Conclusions

A novel dynamic update framework for epileptic seizure prediction system has been proposed, in which the prediction model can be updated and kept fresh. The framework utilizes Mahalanobis distance as the distance matric. Two sample pools filled with preictal samples and interictal samples, respectively, are constructed. Through the judgment of missing alarm and false alarm, the two sample pools are updated and so is the prediction model.

In order to evaluate the performance of the system proposed, careful comparison experiments on the Freiburg database are carried out. Compared to the system without model update, our method is more effective. Under a satisfying sensitivity, the false-positive rate can be as low as 0.04 h^−1^, with the performance index being 0.91. The results clearly indicate that the proposed system can keep fresh at all times. Along with the update of sample pools, the prediction model is updated to be more effective than the early one. In addition, the usage of the multichannel feature extraction method based on HHT and ELM can extract the effective features to distinguish the preictal and interictal states. The whole system is significantly helpful for the exploitation of online portable devices.

## Figures and Tables

**Table 1:** Comparison of no update and dynamic update for the model.

Patient	No update for the model					Dynamic update for the model				
	Sensitivity (%)	Advance times (min)			False-positive rate (h^−1^)	Sensitivity (%)	Advance times (min)			False-positive rate (h^−1^)
		1	2	3			1	2	3	
4	100	79.1	58.7	62.6	0.03	100	79.0	56.2	63.5	0.00
5	66.7	0	24.3	43.8	0.00	66.7	17.5	0	22.0	0.03
9	100	67.8	59.2	81.3	0.00	100	67.8	59.2	81.3	0.00
10	100	18.4	27.3	76.0	0.00	100	20.3	27.1	76.4	0.07
16	66.7	73.3	10.9	0	0.03	66.7	45.0	23.8	0	0.00
17	33.3	108.3	0	0	0.38	66.7	52.8	0	33.3	0.14
18	100	38.3	63.3	93.8	0.11	100	38.3	68.5	93.7	0.00
20	66.7	0	26.9	36.9	0.17	66.7	0	30.9	37.5	0.12
21	100	108.2	102.5	76.0	0.21	100	107.2	103.0	61.5	0.00

**Table 2:** Comparison of different methods.

Items	No update for the model	Dynamic update for the model
Mean sensitivity (%)	81.5	85.2
Mean advance time (min)	49.5	46.9
Mean false-positive rate (h^−1^)	0.10	0.04
Performance index *P *	0.86	0.91

**Table 3:** Patients' characteristics.

Patient	Sex	Age	Seizure type	H/NC	Origin	Electrodes	Number of seizures	Interictal (h)
4	f	26	SP, CP, GTC	H	Temporal	d, g, s	5	24
5	f	16	SP, CP, GTC	NC	Frontal	g, s	5	24
9	m	44	CP, GTC	NC	Temporal/occipital	g, s	5	24
10	m	47	SP, CP, GTC	H	Temporal	d	5	24
16	f	50	SP, CP, GTC	H	Temporal	d, s	5	24
17	m	28	SP, CP, GTC	NC	Temporal	s	5	24
18	f	25	SP, CP	NC	Frontal	s	5	25
20	m	33	SP, CP, GTC	NC	Temporal/parietal	d, g, s	5	26
21	m	13	SP, CP	NC	Temporal	g, s	5	24

Seizure types and location: simple partial (SP), complex partial (CP), generalized tonic-clonic (GTC), hippocampal (H), and neocortical (NC). Electrodes: depth (d), grid (g), and strip (s). Five seizures and at least 24 h of interictal EEG data for every patient were analyzed.

## Appendix

Table 3 lists the characteristics of the 9 patients in the Freiburg database [42], including the descriptions of their sex (“f” for female and “m” for male), age, seizure type (“SP” for simple partial, “CP” for complex partial, and “GTC” for generalized tonic-clonic), seizure location (“H” for hippocampal and “NC” for neocortical), seizure origin, seizure number, electrodes (“d” for depth, “g” for grid, and “s” for strip), and interictal length.

## References

[1] Fisher RS, van Emde Boas W, Blume W (2005). Epileptic seizures and epilepsy: definitions proposed by the International League Against Epilepsy (ILAE) and the International Bureau for Epilepsy (IBE). *Epilepsia*.

[2] Duncan JS, Sander JW, Sisodiya SM, Walker MC (2006). Adult epilepsy. *The Lancet*.

[3] Mormann F, Andrzejak RG, Elger CE, Lehnertz K (2007). Seizure prediction: the long and winding road. *Brain*.

[4] Lopes da Silva FH (2008). The impact of EEG/MEG signal processing and modeling in the diagnostic and management of epilepsy. *IEEE Reviews in Biomedical Engineering*.

[5] Xingyuan W, Chao L (2006). Researches on chaos phenomenon of EEG dynamics model. *Applied Mathematics and Computation*.

[6] He B, Yang L, Wilke C, Yuan H (2011). Electrophysiological imaging of brain activity and connectivity—challenges and opportunities. *IEEE Transactions on Biomedical Engineering*.

[7] Chua KC, Chandran V, Rajendra Acharya U, Lim CM (2009). Analysis of epileptic EEG signals using higher order spectra. *Journal of Medical Engineering and Technology*.

[8] Sabesan S, Chakravarthy N, Tsakalis K, Pardalos P, Iasemidis L (2009). Measuring resetting of brain dynamics at epileptic seizures: application of global optimization and spatial synchronization techniques. *Journal of Combinatorial Optimization*.

[9] Rabbi AF, Aarabi A, Fazel-Rezai R Fuzzy rule-based seizure prediction based on correlation dimension changes in intracranial EEG.

[10] Li X, Ouyang G (2006). Nonlinear similarity analysis for epileptic seizures prediction. *Nonlinear Analysis, Theory, Methods and Applications*.

[11] Chisci L, Mavino A, Perferi G (2010). Real-time epileptic seizure prediction using AR models and support vector machines. *IEEE Transactions on Biomedical Engineering*.

[12] Aarabi A, Fazel-Rezai R, Aghakhani Y EEG seizure prediction: measures and challenges.

[13] Mormann F, Lehnertz K, David P, E. Elger C (2000). Mean phase coherence as a measure for phase synchronization and its application to the EEG of epilepsy patients. *Physica D: Nonlinear Phenomena*.

[14] Han M, Wang M-H, Hong X-J, Han J (2012). Epileptic seizure prediction based on probabilistic discriminative extreme leaning machine. *Chinese Journal of Biomedical Engineering*.

[15] Wang L, Wang C, Fu F (2011). Temporal lobe seizure prediction based on a complex Gaussian wavelet. *Clinical Neurophysiology*.

[16] Mormann F, Kreuz T, Andrzejak RG, David P, Lehnertz K, Elger CE (2003). Epileptic seizures are preceded by a decrease in synchronization. *Epilepsy Research*.

[17] Andrzejak RG, Chicharro D, Lehnertz K, Mormann F (2011). Using bivariate signal analysis to characterize the epileptic focus: the benefit of surrogates. *Physical Review E. Statistical, Nonlinear, and Soft Matter Physics*.

[18] Pabel R, Koch R, Jager G, Kunoth A (2010). Fast empirical mode decompositions of multivariate data based on adaptive spline-wavelets and a generalization of the Hilbert-Huang-Transformation (HHT) to arbitrary space dimensions. *Advances in Adaptive Data Analysis*.

[19] Huang NE, Shen Z, Long SR (1998). The empirical mode decomposition and the Hubert spectrum for nonlinear and non-stationary time series analysis. *Proceedings of the Royal Society A: Mathematical, Physical and Engineering Sciences*.

[20] Sun J, Hong X, Tong S (2012). Phase synchronization analysis of eeg signals: an evaluation based on surrogate tests. *IEEE Transactions on Biomedical Engineering*.

[21] Pereda E, Quiroga RQ, Bhattacharya J (2005). Nonlinear multivariate analysis of neurophysiological signals. *Progress in Neurobiology*.

[22] Huang G-B, Zhu Q-Y, Siew C-K (2006). Extreme learning machine: theory and applications. *Neurocomputing*.

[23] Liu B, Yan L, Li L, Wang W Comparing study of nonlinear model for epileptic preictal prediction.

[24] Liang N-Y, Huang G-B, Saratchandran P, Sundararajan N (2006). A fast and accurate online sequential learning algorithm for feedforward networks. *IEEE Transactions on Neural Networks*.

[25] Zhao J, Wang Z, Park DS (2012). Online sequential extreme learning machine with forgetting mechanism. *Neurocomputing*.

[26] Cordeiro de Amorim R, Mirkin B (2012). Minkowski metric, feature weighting and anomalous cluster initializing in K-Means clustering. *Pattern Recognition*.

[27] Gu F, Liu D, Wang X Semi-supervised weighted distance metric learning for kNN classification.

[28] Xiang S, Nie F, Zhang C (2008). Learning a Mahalanobis distance metric for data clustering and classification. *Pattern Recognition*.

[29] Xing EP, Ng AY, Jordan MI, Russell S (2003). Distance metric learning with application to clustering with side-information. *Advances in Neural Information Processing Systems*.

[30] Schultz M, Joachims T (2004). Learning a distance metric from relative comparisons. *Advances in Neural Information Processing Systems*.

[31] Zhang J, Yan R On the value of pairwise constraints in classification and consistency.

[32] Sonnum S, Thaithieng S, Ano S, Kusolchu K, Kerdprasop N Approximate web database search based on Euclidean distance measurement.

[33] Hori M, Ueda M, Iwata A (2006). Stochastic computing chip for measurement of Manhattan distance. *Japanese Journal of Applied Physics 1*.

[34] Kløve T, Lin T-T, Tsai S-C, Tzeng W-G (2010). Permutation arrays under the Chebyshev distance. *IEEE Transactions on Information Theory*.

[35] Ren Y, Liu X, Liu W (2012). DBCAMM: a novel density based clustering algorithm via using the Mahalanobis metric. *Applied Soft Computing Journal*.

[36] Lehnertz K, Bialonski S, Horstmann M-T (2009). Synchronization phenomena in human epileptic brain networks. *Journal of Neuroscience Methods*.

[37] Rilling G Empirical Mode Decomposition. http://perso.ens-lyon.fr/patrick.flandrin/emd.html.

[38] Bai D-M, Qiu T-S, Bao H-P (2006). A new epileptic prediction method based on EMD and sample entropy. *Chinese Journal of Biomedical Engineering*.

[39] Niang O, Delechelle É, Lemoine J (2010). A spectral approach for sifting process in empirical mode decomposition. *IEEE Transactions on Signal Processing*.

[40] Rilling G, Flandrin P, Goncalves P On empirical mode decomposition and its algorithms.

[41] Chen D, Li D, Xiong M, Bao H, Li X (2010). GPGPU-aided ensemble empirical-mode decomposition for EEG analysis during anesthesia. *IEEE Transactions on Information Technology in Biomedicine*.

[42] Maiwald T, Winterhalder M, Aschenbrenner-Scheibe R, Voss HU, Schulze-Bonhage A, Timmer J (2004). Comparison of three nonlinear seizure prediction methods by means of the seizure prediction characteristic. *Physica D: Nonlinear Phenomena*.

[43] Huang GB MATLAB Codes of ELM Algorithm. http://www3.ntu.edu.sg/home/egbhuang/elm_codes.html.

[44] Winterhalder M, Maiwald T, Voss HU, Aschenbrenner-Scheibe R, Timmer J, Schulze-Bonhage A (2003). The seizure prediction characteristics: a general framework to assess and compare seizure prediction methods. *Epilepsy and Behavior*.

[45] Feldwisch-Drentrup H, Schelter B, Jachan M, Nawrath J, Timmer J, Schulze-Bonhage A (2010). Joining the benefits: combining epileptic seizure prediction methods. *Epilepsia*.

[46] Ouyang G, Li X, Li Y, Guan X (2007). Application of wavelet-based similarity analysis to epileptic seizures prediction. *Computers in Biology and Medicine*.

[47] Winterhalder M, Schelter B, Maiwald T (2006). Spatio-temporal patient-individual assessment of synchronization changes for epileptic seizure prediction. *Clinical Neurophysiology*.

[48] Aarabi A, He B (2012). A rule-based seizure prediction method for focal neocortical epilepsy. *Clinical Neurophysiology*.

[49] Williamson JR, Bliss DW, Browne DW Epileptic seizure prediction using the spatiotemporal correlation structure of intracranial EEG.

[50] Wang N, Lyu MR Exploration of instantaneous amplitude and frequency features for epileptic seizure prediction.

